# Pseudofungi in an Immunocompromised Patient with Breast Cancer and COVID-19

**DOI:** 10.1155/2020/1849250

**Published:** 2020-10-29

**Authors:** Nadeem Bilani, Leah Elson, Diane Carlson, Elizabeth Blessing Elimimian, Zeina Nahleh

**Affiliations:** ^1^Department of Hematology-Oncology, Maroone Cancer Center, Cleveland Clinic Florida, Weston, FL, USA; ^2^Department of Pathology, Cleveland Clinic Florida, Weston, FL, USA

## Abstract

Herein, we present a case of a male patient with breast cancer and a recent history of COVID-19 pneumonia, diagnosed with pseudofungi on pathological examination of lymph nodes after mastectomy. Pseudofungi are septate hyphae-like structures that morphologically mimic fungal elements despite the absence of true mycosis and thus predispose to overtreatment if not properly identified. We report a review of similar cases involving this diagnostic mimicker in the literature.

## 1. Background

Fungal infections can be diagnosed via identification of hyphae or yeast structures in tissue specimens [1]. Pseudofungi, which are septate, hyphae-like structures mimicking the morphology of true mycosis [2], present a unique diagnostic challenge. Special stains, such as Grocott's methenamine silver (GMS) or periodic acid–Schiff (PAS), bind specifically to fungal components and can thus help to more definitively rule out pseudofungi.

## 2. Case Report

An elderly Black male with ER+/PR+/HER2 negative, stage IIIB cT4N2M0 invasive ductal carcinoma presented to the Emergency Department with bleeding from the left breast for several days due to fungating breast cancer. A complete blood count revealed anemia (hemoglobin, 9.3 g/dL), 578,000 platelets/uL, and normal total white blood cell and absolute neutrophil counts. The patient was on anticoagulation therapy with 15 mg twice-daily rivaroxaban after being diagnosed twenty days prior with a bilateral pulmonary embolism (PE) resulting in lung infarcts in the right middle and left lower lobes. These events followed a recent history of COVID-19 infection causing bilateral interstitial pneumonia complicated by sepsis, treated with hydroxychloroquine and azithromycin. This acute infectious history resulted in a 3-week delay of the patient's neoadjuvant chemotherapy with paclitaxel. Due to concerns for disease progression with further delay, he was referred for surgical management. The patient received a left-modified radical mastectomy with partial excision of the pectoralis major muscle and axillary lymphadenectomy.

**Figure 1:**
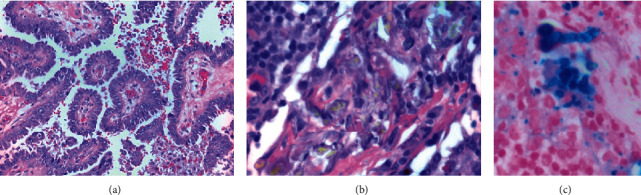
H&E staining of (a) primary breast carcinoma under high magnification demonstrating papillary architecture and nuclear pleomorphism. (b) Lymph node with what appears to be branching fungal forms. (c) Perl's iron stain of lymph node under high power showing septated, hyphae-like structures.

Pathology after mastectomy revealed a 15 cm mass fungating through the skin, with papillary architecture and nuclear pleomorphism (Figure 1(a)), and eleven out of twenty-eight lymph nodes were positive for malignant involvement. Additionally, at least twenty lymph nodes demonstrated subcapsular and intrasinusoidal refractile birefringent crystal-like structures. On staining with hematoxylin and eosin (H&E), they were morphologically suggestive of infection with *Aspergillus* (Figure 1(b)). There were no necrotizing granulomas in any lymph nodes; however, there were areas of foreign body giant cell reaction and extensive hemosiderin pigment deposition.

After consulting the infectious disease specialist, the patient was given two doses of voriconazole and underwent multiple diagnostic tests, including CXR, CT of the chest and sinus, UA, aspergillosis galactomannan, fungal blood cultures, and procalcitonin. The results of all testing were unremarkable. He was febrile only in one temperature reading during his postsurgical stay, likely due to atelectasis, and the patient denied any other symptoms.

Further evaluation of lymph node blocks using GMS, PAS, and Gram stains was negative for microorganisms. Iron staining, however, demonstrated an abundance of deposition, including the septated hyphae-like structures identified on H&E (Figure 1(c)). Initial findings on lymph nodes were thus attributed to pseudofungi. With true mycosis ruled out, antifungals were discontinued, and focus was switched to balancing the patient's anticoagulation status. In the weeks that followed, the patient remained afebrile, and there were no other signs or symptoms of infection.

## 3. Discussion

We, hereby, describe a case of pseudofungi in an immunocompromised male with breast cancer receiving chemotherapy until a recent COVID-19 infection. Understanding diagnostic mimickers is an essential component of clinical practice, especially in patients with cancer whose immunocompromised state may contribute towards blunted or atypical disease presentations. Overtreatment not only contributes to waste in healthcare spending but also exposes patients to unnecessary risks of prolonged hospital stays and treatment toxicities.

There are few published case reports of pseudofungi [1–5]. Despite the morphological similarity between true mycosis and pseudofungi, a number of notable differences can be made on histology. True fungal infection usually presents with necrosis and granulomatous inflammation [1]. Such an inflammatory response was absent in this case, but thought to potentially be attributed to the patient's immunocompromised status [6]. Additionally, pseudofungi typically stain yellow-brown on H&E, while hyaline fungi such as *Aspergillus* are colorless and stain basophilic [1]. The use of additional stains, as performed in this case, can help clarify the diagnostic picture. As pseudofungi commonly occur in settings of hemorrhage, during which iron and calcium salts may be deposited in tissue, they will stain positive for Perl's iron stain, while being negative for stains that can typically confirm true mycoses, e.g., GMS or PAS [4].

This case also exemplifies the oftentimes precarious balancing act of managing the anticoagulation status of cancer patients in this new COVID-19 era. Cancer itself lends to hypercoagulability [7]. When combined with this patient's acute COVID-19 infection, which has also been associated with venous thromboembolic events [8], he developed PE. Anticoagulation therapy likely exacerbated bleeding from his fungating breast cancer wound, making it necessary to manage surgically sooner than anticipated. Pseudofungi in the setting of hemorrhage should be considered a potential confounding diagnosis to avoid unnecessary and lengthy treatment of fungal infection.

## Data Availability

Due to the nature of this research, the patient did not agree for identifying data to be shared publicly, so supporting data are not available. This case has been anonymized by the International Committee of Medical Journal Editors (ICMJE) standards.
